# A reliable nomogram model for predicting esophageal stricture after endoscopic submucosal dissection

**DOI:** 10.1097/MD.0000000000028741

**Published:** 2022-02-04

**Authors:** Guodong Yang, Zhao Mu, Ke Pu, Yulin Chen, Luoyao Zhang, Haiyue Zhou, Peng Luo, Xiaoying Zhang

**Affiliations:** a Department of Gastroenterology, The Affiliated Hospital of North Sichuan Medical College, Sichuan, China.; b Teaching, and Research Section of Parasitology, School of Basic Medicine, North Sichuan Medical College, Nanchong, Sichuan, China.

**Keywords:** endoscopic submucosal dissection, esophageal stenosis, nomogram, predict, risk-factors

## Abstract

Currently, endoscopic submucosal dissection (ESD) has gradually become the diagnosis and treatment of choice for initial esophageal cancer. However, the formation of esophageal stricture after ESD is one of its important complications. In this paper, we intend to identify the risk factors of esophageal stricture to develop a nomogram model to predict the risk of esophageal stricture and validate this model.

A total, 159 patients were included in this study, including 21 patients with esophageal stenosis. Multivariate analysis showed that age greater than 60 years, high neutrophil-to-lymphocyte ratio, the extent of esophageal mucosal defect greater than 1/2, and postoperative pathological type of early esophageal squamous cell carcinoma were independent risk factors for predicting esophageal stricture. We constructed a nomogram model to predict esophageal stenosis by these 4 independent predictors.

The prediction performance of the model was verified by the area under the receiver operating characteristic curve, the area under the receiver operating characteristic curve of the model was 0.889, and the sensitivity and specificity were 80.00% and 91.28%, respectively, indicating that the prediction performance of the model was good; The calibration curve constructed by internal cross-validation suggested that the predicted results of the nomogram agreed well with the actual observed values.

The nomogram model has a high accuracy for predicting esophageal stricture after esophageal ESD and is extremely important to reduce or avoid the occurrence of esophageal stricture. But it needs more external and prospective validation.

## 1 Introduction

Esophageal cancer is one of the most common cancers worldwide and ranks sixth in cancer mortality worldwide,^[1,2]^ and esophageal squamous cell carcinoma (ESCC) is the predominant histological type. Early-onset symptoms of esophageal cancer are generally atypical, and late stages are often diagnosed and detected due to swallowing discomfort or difficulty, with poor prognosis and poor overall survival.^[3,4]^ At present, the treatment of early esophageal cancer is often based on endoscopic submucosal dissection (ESD) resection, but postoperative complications such as bleeding, perforation, stenosis, infections may occur,^[5]^ of which the formation of esophageal stricture often makes patients have dysphagia, eating obstruction symptoms, seriously affecting the quality of life of patients.

The nomogram is a statistically-based prediction model for predicting the probability of clinical outcome occurrence.^[6]^ At present, nomograms have been widely used in the study of diseases,^[7–9]^ and the application of this model plays an important role in predicting the individualized evaluation of patients, the occurrence of diseases, and the survival prognosis. However, there are still no relevant reports using nomograms to predict the occurrence of esophageal stricture after ESD.

Therefore, to improve the understanding of esophageal stricture and take corresponding preventive measures, this paper develops a nomogram model to predict the risk of esophageal stricture by identifying the risk factors of stricture after ESD in patients with early esophageal cancer and validates this model.

## 2 Patients and methods

From January 2017 to June 2021, the database of electronic medical records and endoscopy centers of the Affiliated Hospital of North Sichuan Medical College was retrospectively searched. After ESD preoperative biopsy confirmed low-grade intraepithelial neoplasia, high-grade intraepithelial neoplasia or ESCC, all patients underwent endoscopic ultrasound or pathological diagnosis after ESD confirmed that the lesion did not exceed SM1, and CT excluded distant metastasis, and 159 patients were finally included. All ESD procedures were performed by a senior endoscopist, and all lesions could be divided into multiple resections or multiple resections, but all were complete resections, resection of the lateral and basal margins without tumor. The present study was approved by the ethics committee of The Affiliated Hospital of North Sichuan Medical College, and the need for obtaining informed consent from patients was waived due to the retrospective nature of the study.

### 2.1 The inclusion criteria

1. Preoperative comprehensive imaging examination to rule out peripheral lymph nodes and distant metastasis;
2. Endoscopic biopsy was performed, and the pathological results of the preoperative biopsy were early esophageal cancer or precancerous lesions;
3. Preoperative endoscopic ultrasonography was performed to rule out the situation that the depth of tumor invasion exceeded the mucosal layer or surrounding lymph node metastasis;
4. Pathological diagnosis after ESD revealed that the depth of tumor invasion did not exceed 200 μm in the submucosa or the upper third of the submucosal; and
5. Patients without serious underlying diseases.

### 2.2 The exclusion criteria

1. Patients with severe heart, liver, lung, kidney, and blood system diseases;
2. Patients with mental disorders or severe mental disorders;
3. Patients with malignant tumors or distant metastasis;
4. Patients with leiomyoma or papilloma after ESD;
5. Patients with other tumors, previous history of radiotherapy and surgery;
6. Patients with other surgical dissection of esophageal mucosal lesions such as MBM and argon plasma coagulation; and
7. Patients with ESD in other hospitals.

### 2.3 ESD method

After satisfactory routine anesthesia, the patient was placed in left lateral decubitus position, transparent cap with gastroscope band was routinely inserted, 2% Lugol iodine solution was used to stain the lesion to ensure clear margin, NBI combined with magnifying endoscopy was performed to observe mucosal IPCL when necessary, the lesion was marked under magnifying endoscopy, methylene blue, glycerin fructose, epinephrine, and sodium hyaluronate were injected submucosally, after repeated injection of the bulge, the circular incision was performed with dual knife, layer-by-layer dissection was performed with dual knife, intermittent injection separation was performed, supplemented by hot hemostatic forceps for hemostasis, until the lesion was completely dissected, and the specimen was recovered and submitted for examination. After returning to the ward after surgery, all patients were treated with fasting, omeprazole (40 mg bid) for acid suppression, selective oral steroids (30 mg QD), preventive anti-infection, and fluid support, and all hospitalized patients were discharged 3 to 5 days after ESD.

### 2.4 Pathological evaluation

The post-ESD specimen was immersed in a 10% formalin container and immediately sent to the pathology department, and the postoperative pathological assessment was completed by an experienced pathologist. The pathological diagnostic criteria followed the World Health Organization criteria, and the pathological diagnosis was classified as precancerous lesions (low-grade intraepithelial neoplasia, high-grade intraepithelial neoplasia), well-differentiated squamous cell carcinoma, moderately differentiated squamous cell carcinoma, or poorly differentiated squamous cell carcinoma.^[10]^ According to WHO criteria,^[11]^ the depth of lesion invasion is divided into M phase of cumulative mucosal layer and SM phase of cumulative submucosal layer, in which M phase is further divided into M1 involving mucosal epithelial layer, M2 involving lamina propria, and M3 involving mucosa; the submucosal layer is divided into upper, middle and lower 3 layers, and SM phase is divided into SM1, SM2, and SM3 through the depth of lesion invasion.

### 2.5 Follow-up

Patients were instructed to undergo gastroscopy at 3 months, 6 months, and 12 months after ESD, and again at 1 or 2 years thereafter. During endoscopic follow-up, special attention was paid to the site of suspected residual or recurrent resection, and the surgical resection site was evaluated by screenshot. The postoperative esophageal stricture was defined using Katada et al^[12]^ criteria, that is, esophageal stricture was diagnosed when the endoscope (11 mm in diameter) could not pass through.

### 2.6 Risk factors

The related factors of esophageal stricture and nonesophageal stricture were compared, including basic characteristics of patients, lesion factors, and surgery-related factors. Patient factors included age, gender, family history, smoking and alcohol history, preoperative neutrophil-to-lymphocyte ratio (NLR), platelet-to-lymphocyte ratio, and lympho-monocyte ratio. Lesion-related factors include: lesion location, depth of Infiltration, macroscopic type, postoperative pathological type (PPT), etc; surgery-related factors include: longitudinal and transverse long diameter of mucosal defects, the circumferential ratio of the mucosal defect, en bloc resection, and whether oral steroids are taken after surgery. The location of esophageal mucosal lesions was divided into 3 segments: upper, middle, and lower,^[13]^ in which the upper finger was 15 - 24 cm from the incisors, the middle finger was 24 to 32 cm from the incisors, and the lower finger was 32 to 40 cm. According to the 2002 Paris classification,^[14]^ the macroscopic types of lesions were divided into “elevated type (type I), superficial elevated type (IIa), superficial flat type (IIb), superficial depressed type (IIc), and depressed type (type III)”. The circumferential ratio of the mucosal defect after ESD was divided into 4 groups (12): less than one-quarter (<1/4), one-quarter to one-half (1/4–1/2), one-half to three-quarters (1/2–3/4), and more than three-quarters (>3/4).

### 2.7 Statistical analysis

Comparisons of categorical variables were performed with Fisher exact test and chi-square test. The *t* test was used for the analysis of continuous variables, and the results were expressed as mean ± standard deviation. Statistical analyses were performed using SPSS software and R software (version 4.2.0). *P*-value, odds ratio, and 95% confidence interval (CI) were used to describe all risk factors of esophageal stenosis in this study. Univariate analysis was used to include predictors with *P* < .05 in binary logistic regression analysis, and finally, independent predictors of esophageal stenosis were obtained. The nomogram for predicting esophageal stricture was successfully established based on its results independent variable regression coefficient and R software, and the discrimination and calibration of the prediction model were assessed. Discrimination of the predictive model refers to the ability to discriminate between stenotic and nonstenotic patients, and calibration refers to the agreement between the predicted and observed probabilities. Discrimination is often assessed using the area under receiver operating characteristic curve (AUC). In this paper, receiver operating characteristic curves were drawn by SPSS for internal validation of nomograms to determine the accuracy of the prediction model. Calibration is the evaluation of the model by drawing a calibration curve. In this paper, we perform 1000 sampling tests on the results through R and successfully draw the calibration curve, to evaluate that the model prediction results are consistent with the actual observed results.

## 3 Results

### 3.1 Patient demographics

Of the 284 patients treated with esophageal ESD, patients with tumors exceeding SM1, postoperative pathological confirmation of leiomyoma, papilloma, tubular adenoma, and missing follow-up were excluded. Therefore, 159 patients were included in the study. Of the 159 patients, 91 were male (57.23%). The mean age was 63.11 years (range, 36–79 years). During the follow-up period, there was no recurrence or metastasis in any of the therapeutic resection cases, and 21 patients (13.21%) developed esophageal stricture after surgery. The baseline characteristics, lesion characteristics, pathology after ESD, and stenosis of the patients are detailed in Table 1.

**Table 1:** Baseline characteristics of the patients.

	Postoperative stricture	No postoperative stricture	*P*-value
Number	21	138	
Sex, male/female, n	12/9	79/59	.993
Age, n			
>60	2	47	.038
≤60	19	91	
Family history, n	3	9	.222
Smoke, n	9	46	.395
Drink, n	4	25	.940
Complications, n			
Hypertension	3	22	.846
Diabetes	1	8	.849
Lesion location, n			.454
Upper	4	14	
Middle	15	104	
Lower	2	20	
NLR, n			.004
≤2.283	3	72	
>2.283	18	66	
PLR, n			.211
≤102.41	8	73	
>102.41	13	65	
LMR, n			.937
≤4	11	71	
>4	10	67	
Longitudinal length, mean ± SD, cm	5.014 ± 1.492	3.471 ± 1.649	.001
Transverse diameter, mean ± SD, cm	3.171 ± 0.89	2.317 ± 1.143	.01
CRMD, n			.003
<1/4	0	20	
1/4∼1/2	4	78	
1/2∼3 /4	9	27	
>3/4	8	13	
En bloc resection, n	17	120	.638
Steroid, n	13	35	.001
PPT, n			.013
LGIN	3	41	
HGIN	7	68	
ESCC	11	29	
Depth of infiltration, n			.027
<m2	12	110	
≥m2	9	28	

CRMD = the circumferential ratio of the mucosal defect, ESCC = esophageal squamous cell carcinoma, HGIN = high-grade intraepithelial neoplasia, LGIN = low-grade intraepithelial neoplasia, LMR = lympho-monocyte ratio, MLR = monocyte-to-lymphocyte ratio, NLR = neutrophil-to-lymphocyte ratio, PLR = platelet-to-lymphocyte ratio, PPT = postoperative pathological type.

### 3.2 Nomogram development

**Figure 1:**
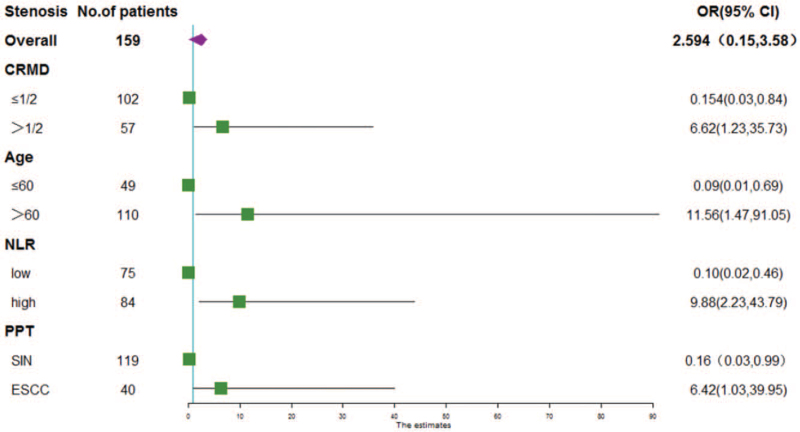
Forest plot of each predictor. The left column lists the names of the predictors. The OR for each of these studies is represented by a square, and CIs are represented by horizontal lines. CI = confidence interval, CRMD = the circumferential ratio of the mucosal defect, ESCC = esophageal squamous cell carcinoma, OR = odds ratio, NLR = neutrophil-to-lymphocyte ratio, PPT = postoperative pathological type, SIN = squamous intraepithelial neoplasia.

**Figure 2:**
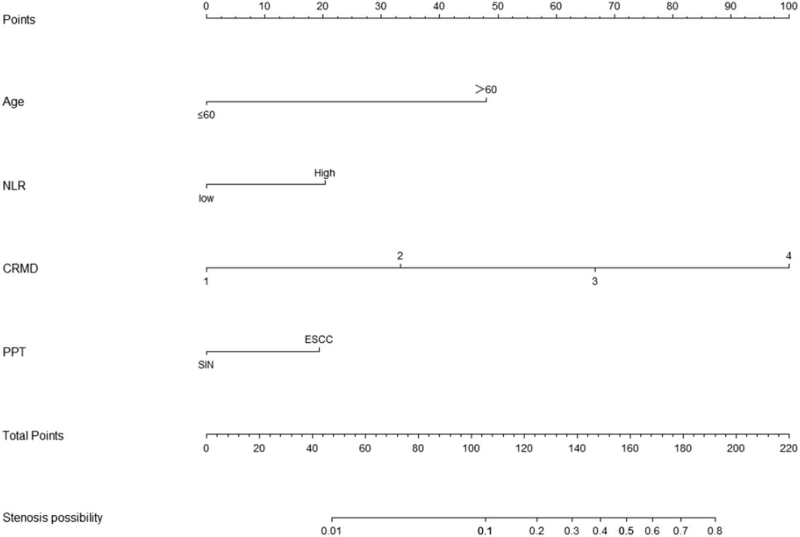
Risk-prediction nomogram for patients with postoperative esophageal ESD. CRMD = the circumferential ratio of the mucosal defect, ESCC = esophageal squamous cell carcinoma, NLR = neutrophil-to-lymphocyte ratio, PPT = postoperative pathological type.

In the stenosis group (n = 21) and the nonstenosis group (n = 138), we first performed a univariate analysis of the related factors, and the results are shown in Table 1: there were no significant differences in gender, family history, lesion location, and macroscopic type, preoperative platelet-to-lymphocyte ratio and lympho-monocyte ratio, smoking history, drinking history, hypertension and diabetes history, and the number of resections between the 2 groups (*P* > .05). However, it was related to age, preoperative NLR, whether steroids were used, longitudinal diameter of the resected specimen, the transverse diameter of the resected specimen, the extent of esophageal mucosal defect, PPT, and depth of infiltration (*P* < .05). Statistically significant variables were selected from univariate analysis and included in multivariate analysis, and the results are shown in Table 2: age greater than 60 years, high NLR, esophageal mucosal defect range more than 1/2, and PPT of early ESCC were independent risk factors for stenosis after esophageal ESD (*P* < .05). The probability of postoperative esophageal stenosis in patients older than 60 years was 11.56 times higher than that in patients younger than 60 years (95% CI, 1.47–91.05; *P* < .05). The probability of postoperative esophageal stenosis in patients with high NLR was 9.88 times higher than that in patients with low NLR (95% CI, 2.23–43.79; *P* < .05). Patients with esophageal mucosal defects more than 1/2 had a 6.62-fold higher probability of postoperative esophageal stenosis than patients with esophageal mucosal defects less than or equal to 1/2 (95% CI, 1.23–35.73; *P* < .05). The probability of esophageal stenosis in patients with early ESCC was 6.42 times higher than that in patients with squamous intraepithelial neoplasia (95% CI, 1.03–39.95; *P* < .05). The results of multivariate analysis are shown in the forest plot (Fig. 1). We performed a collinearity analysis of the above independent risk factors, and the variance inflation factors were 1.019, 1.007, 1.059, and 1.070, respectively, which indicated that there was no multicollinearity among the 4 independent risk factors. Based on the results of multivariate analysis, we developed an individualized prediction model containing 4 independent risk factors for esophageal stricture after ESD, the nomogram model (Fig. 2). The total risk score was determined by obtaining the point for each factor of interest, adding the scores of all variables, and directly obtaining the probability of esophageal stricture occurrence, and if the probability was greater than 50%, we predicted that the patient would develop esophageal stricture.

**Table 2:** Multivariate logistic regression model.

Variables	OR	95% CI	*P*-value
Age			
≤60	1		
>60	11.562	1.468∼91.054	.02
NLR			
≤2.283	1		
>2.283	9.876	2.227∼43.788	.003
Longitudinal length	1.12	0.701∼1.790	.635
Transverse diameter	1.354	0.679∼2.698	.389
Steroid			
No	1		
Yes	1.071	0.24∼4.785	.929
CRMD			
≤1/2	1		
>1/2	6.622	1.227∼35.732	.028
PPT			
SIN	1		
ESCC	6.423	1.033∼39.95	.046
Depth of infiltration			
<m2	1		
≥m2	3.033	0.457∼20.146	.251

CI = confidence interval, CRMD = the circumferential ratio of the mucosal defect, ESCC = esophageal squamous cell carcinoma, NLR = neutrophil-to-lymphocyte ratio, OR = odds ratio, PPT = postoperative pathological type, SIN = squamous intraepithelial neoplasia.

### 3.3 Nomogram validation

**Figure 3:**
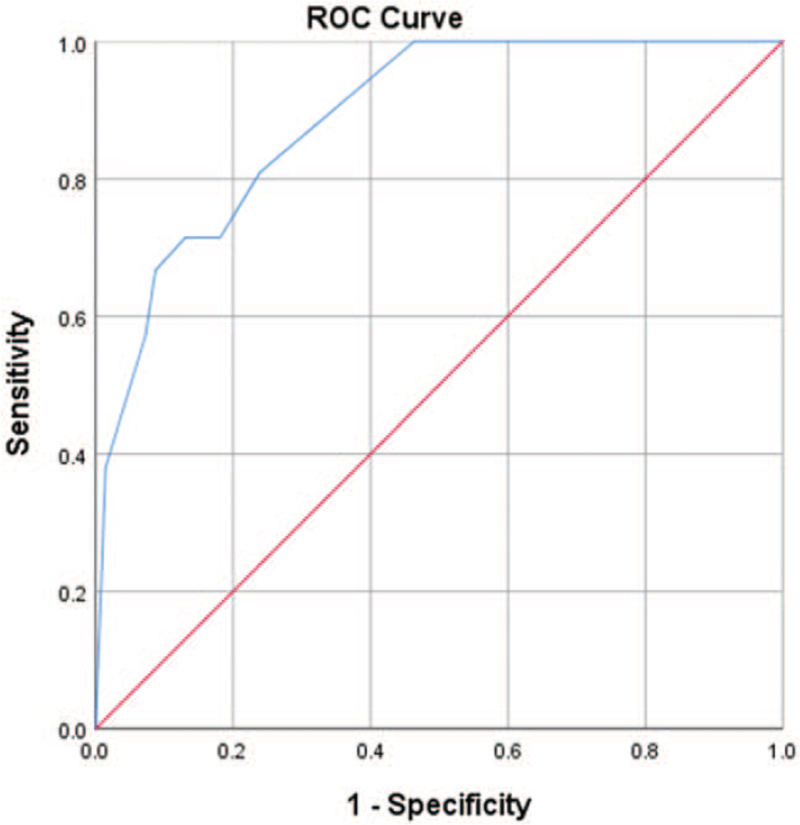
ROC curve for validating the discrimination power of the nomogram. ROC = receiver operating characteristic curve.

**Figure 4:**
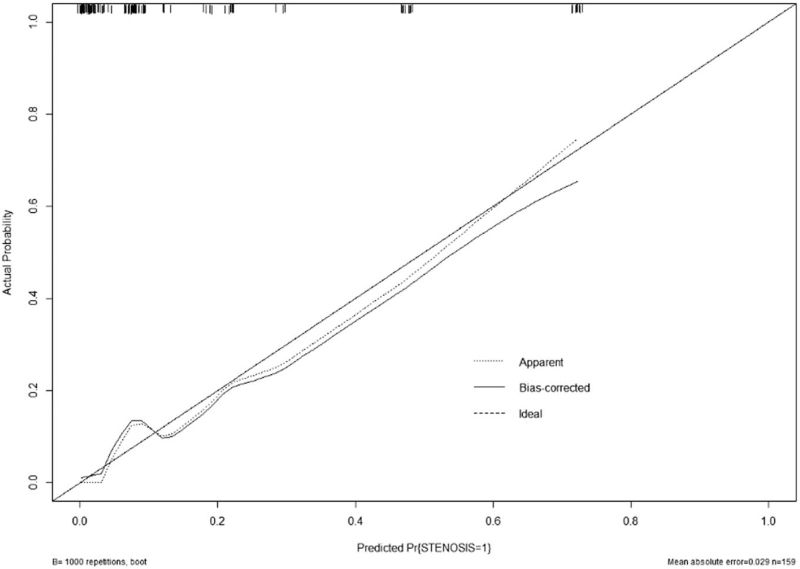
The calibration curve for the test accuracy of the nomogram.

In this paper, we validate the model by discriminating and calibrating. In this study, the discrimination estimation was performed by constructing a nomogram model with 4 statistically significant variables in the results of multivariate analysis, and the results showed (Fig. 3) that the AUC was 0.889, and the sensitivity and specificity were 80.00% and 91.28%, respectively, indicating that the discrimination and predictive performance of this model was good. The calibration curve is shown (Fig. 4) that the probability estimation of the nomogram is in good agreement with the actual observed results, indicating that the prediction model is in good agreement.

## 4 Discussion

In recent years, ESD has gradually become the first choice of treatment for precancerous lesions of esophageal cancer and early esophageal cancer, but esophageal stricture, as one of its postoperative complications, greatly affects the prognosis and quality of life of patients. In general, the repair of esophageal mucosal defects after ESD includes 2 forms: 1 is the regeneration of normal epithelial cells around the mucosal defect, and the other is the maturation of granulation tissue into fibrous connective tissue. The surgically wound repair is divided into 3 stages: inflammatory response, epithelial proliferation, and extracellular matrix remodeling.^[16]^ Some experts in the early stage believe that the mechanism of esophageal stenosis after endoscopic treatment of early esophageal mucosal lesions may be delayed mucosal healing,^[12]^ severe inflammatory response and tissue fibrosis in the submucosa, extracellular matrix proliferation, and proper collapse based on abnormal esophageal motility and pathological abnormalities.^[17,18]^ Honda et al^[19]^ speculated through animal experiments that acute inflammation, deep ulcer, and granuloma formation were caused by the wound after early ESD, and granuloma maturation in the submucosa and the process of healing of mucosal defects in the later stage transformed into fibrous connective tissue, fibrous hyperplasia, and reduced elasticity and motility of the esophageal wall by esophageal wall fibrosis may be the causes of esophageal stricture formation. However, Liu et al^[20]^ also pointed out that this is not the main mechanism of the postoperative stricture formation process. From recent studies, the first choice for esophageal stenosis after ESD may be that the lesions in the esophageal mucosal layer themselves promote the immune-inflammatory response, resulting in the accumulation of a large number of inflammatory mediators, fibrocytes, extracellular matrix, etc in the submucosa, followed by a large postoperative wound and aggravated inflammatory response that further affect the esophageal wound healing. With the further extension of time, the granulomas formed by submucosal inflammation gradually mature, myofibroblasts migrate and proliferate, smooth muscle cell fibrosis and a large number of collagen fiber formation, and finally, esophageal stenosis is formed. The mechanism of postoperative esophageal stricture has not been fully elucidated, and further studies are needed in the future.

This study showed that age older than 60 years, high NLR, esophageal mucosal defect range more than 1/2, and PPT of early ESCC were independent risk factors for stenosis after esophageal ESD (*P* < .05). It has been reported that the extent and size of esophageal mucosal dissection are considered reliable risk factors and independent predictors of esophageal stricture.^[12,21–23]^ Katada et al^[12]^ and Ono et al^[23]^ performed a retrospective logistic regression analysis of patients who had undergone EMR or ESD to investigate the occurrence and severity of strictures after EMR and found that the frequency of esophageal strictures with a circumference of more than three-quarters and a longitudinal diameter >30 mm was significantly higher in patients with mucosal defects, thus confirming the relationship between the extent and size of mucosal dissection and esophageal strictures. In this study, esophageal stenosis occurred in 4 patients with esophageal mucosal defect circumference ratio less than 1/2, and multivariate logistic regression results indicated that the extent of esophageal mucosal defect more than 1/2 was considered to be an independent risk factor for esophageal stenosis after ESD, while the longitudinal diameter and transverse diameter size of the resected specimen were not statistically significant in multivariate logistic regression. The results of this study were not completely consistent with previous reports, which were probably caused by a combination of factors such as different study subjects, inconsistent postoperative management of patients, and differences in patient's living habits. Therefore, endoscopists need to take necessary precautions for patients undergoing massive ESD surgery. Few previous studies on risk factors for esophageal stricture have reported the effect of age on esophageal stricture, and the results of this study confirmed that age is also one of the independent risk factors for esophageal stricture. Factors such as increased infection, trace elements and vitamin deficiency related to wound repair, and poor local blood circulation due to low immunity in elderly patients may be the reasons why elderly patients are prone to form stenosis. Therefore, patients older than 60 years undergoing ESD need to focus on postoperative esophageal stenosis and timely preventive measures.

There have been previous reports on the use of inflammatory factors as esophageal tumor markers,^[24]^ but there are few studies on the association between inflammatory factors and esophageal stricture after ESD. In this study, high NLR was an independent risk factor for esophageal stenosis after ESD. The reason for this may be that esophageal mucosal lesions themselves promote the immune-inflammatory response, tumor cells can secrete inflammatory cytokines, resulting in the presence of a large number of inflammatory mediators in the mucosa and submucosa, TNF-α is the earliest and most important inflammatory mediator, can promote the increase of neutrophil count and lymphocyte count, that is, NLR may be increased, IL-6 can stimulate the differentiation of megakaryocytes and platelets and thrombopoietin production, resulting in increased platelet counts in patients.^[25,26]^ A large number of inflammatory factors lead to granuloma gradual maturation, smooth muscle cell fibrosis and a large number of collagen fiber formations, and finally the formation of esophageal stenosis. Therefore, esophageal mucosal lesions promote an immune-inflammatory response resulting in varying proportions of the elevated preoperative neutrophil count, lymphocyte count, and platelet count, while elevated neutrophil count predominates, that is, high preoperative NLR predisposes to esophageal stricture formation.

This study also showed that PPT of early ESCC was closely related to esophageal stricture after ESD. Although in previous reports, there have been no reports of PPT as an independent risk factor for esophageal stricture after ESD. However, esophageal tumors play a crucial role in the tumor microenvironment relative to precancerous lesions. Inflammatory cells and inflammatory factors are important components of the tumor microenvironment, which can promote the activation of tumor cells and stromal cells, and then produce some inflammatory chemokines and cytokines. Inflammatory cells infiltrating in the esophageal epithelium can break through the esophageal basement membrane to reach the esophageal mucosal epithelium, by releasing a large number of inflammatory mediators, resulting in gradual maturation of granulomas, smooth muscle cell fibrosis, and the formation of a large number of collagen fibers, and finally the formation of esophageal stenosis.^[27]^ Second, tumor cells will promote neovascularization, and blood vessels provide oxygen and nutrients for tumor growth, which in turn promotes tumor metastasis,^[28]^ and abundant angiogenesis will be more conducive to inflammatory production after ESD and accelerate scar formation. Previous studies have found that the depth of tissue invasion is an independent risk factor for esophageal stricture,^[15,29]^ and esophageal stricture after ESD is related to the depth and breadth of the lesion. ESCC is invasive relative to squamous intraepithelial neoplasia, and increased depth of invasion leading to complete resection of the lesion after surgery may increase damage to the mucosa or mucosa. However, in this study, the depth of invasion was not associated with esophageal stricture after ESD, which was the same as the results of Miwata et al^[30]^ Our study showed that PPT was the independent risk factor for esophageal stricture after ESD, rather than the depth of tissue invasion, which may be related to tumor tissue secretion of chemokines, and inflammatory factors, promotion of neovascularization, and other microenvironment changes, but the specific mechanism is unclear and needs further study.

This study is the first to establish a nomogram model to predict esophageal stricture after esophageal ESD. The model predicted esophageal stricture with an AUC of 0.889, sensitivity and specificity of 80.00% and 91.28%, respectively, with good prediction results. The nomogram developed in this paper is suitable for in-hospital patients after esophageal ESD and is a free tool beneficial to clinical risk assessment, which helps clinicians pay more attention to patients with higher risk after esophageal ESD and more actively prevent and treat esophageal strictures.

However, this study still has some limitations. The first choice, our study belongs to a small sample as well as a single-center, retrospective design, so the clinical validity of our nomogram needs to be more prospective studies in the future and verified by external data. In addition, this paper did not perform dynamic follow-up of nutritional status in patients after ESD, but this is also a factor affecting the outcome, and relevant studies need to be focused on in the future.

## 5 Conclusion

In this paper, we developed an individualized nomogram model of esophageal stricture after ESD, which is pioneering. Through this prediction model, we can relatively accurately predict the probability of risk of esophageal stenosis in patients, which helps clinicians to have a clearer judgment on whether postoperative stenosis occurs, and takes targeted preventive measures in advance for patients, which plays an important role in reducing or avoiding the occurrence of esophageal stenosis. At the same time, more external and prospective validation is needed in the future.

## Acknowledgments

The authors thank Prof Xiaoying Zhao for the excellent assistance.

## Author contributions

Guodong Yang, Zhao Mu conducted this study design and manuscript drafting. Pu Ke, Yulin Chen, Luoyao Zhang, Haiyue Zhou, Peng Luo contributed to the acquisition of clinical data. Xiaoying Zhang provided the statistical analysis and review this paper. Guodong Yang, Zhao Mu, Pu Ke, Yulin Chen, Luoyao Zhang, Haiyue Zhou, Peng Luo: All authors participated in this study.

**Conceptualization:** Zhao Mu, Xiaoying Zhang.

**Data curation:** Zhao Mu, Yulin Chen, Luoyao Zhang, Haiyue Zhou, Peng Luo.

**Formal analysis:** Guodong Yang, Zhao Mu, Ke Pu, Yulin Chen, Xiaoying Zhang.

**Funding acquisition:** Guodong Yang, Xiaoying Zhang.

**Investigation:** Zhao Mu, Ke Pu, Xiaoying Zhang.

**Methodology:** Ke Pu, Xiaoying Zhang.

**Project administration:** Guodong Yang.

**Resources:** Yulin Chen, Xiaoying Zhang.

**Software:** Zhao Mu.

**Validation:** Xiaoying Zhang.

**Visualization:** Ke Pu, Yulin Chen, Xiaoying Zhang.

**Writing – original draft:** Guodong Yang, Zhao Mu.

**Writing – review & editing:** Xiaoying Zhang.

## References

[1] Arnold M. Global incidence of oesophageal cancer by histological subtype in 2012. Gut 2015;64:381–7. 25320104 10.1136/gutjnl-2014-308124

[2] Murphy G. International cancer seminars: a focus on esophageal squamous cell carcinoma. Ann Oncol 2017;28:2086–93. 28911061 10.1093/annonc/mdx279 PMC5834011

[3] Japan Esophageal Society. Japanese Classification of Esophageal Cancer, 11th ed.: part I. Esophagus 2017;14:01–36. 10.1007/s10388-016-0551-7 PMC5222932 28111535

[4] Bray FFJS. Erratum: global cancer statistics 2018: GLOBOCAN estimates of incidence and mortality worldwide for 36 cancers in 185 countries. CA Cancer J Clin 2020;70:313–1313. 10.3322/caac.21609 32767693

[5] Rizvi QMF. Endoscopic management of early esophagogastric cancer. Surg Oncol Clin N Am 2016;26:179–91. 10.1016/j.soc.2016.10.007 28279463

[6] Bianco FJ. Nomograms, and medicine. Eur Urol 2006;50:884–6. 16973258 10.1016/j.eururo.2006.07.043

[7] Yan C Yang M Huang Y. A Reliable nomogram model to predict the recurrence of chronic subdural hematoma after burr hole surgery. World Neurosurg 2018;118:e356–66. 29969745 10.1016/j.wneu.2018.06.191

[8] Chao Y. Development of a nomogram for the prediction of pathological complete response after neoadjuvant chemoradiotherapy in patients with esophageal squamous cell carcinoma. Dis Esophagus 2016;30:01–8. 10.1111/dote.12519 27868287

[9] Zheng H. Nomogram to predict lymph node metastasis in patients with early oesophageal squamous cell carcinoma. Br J Surg 2018;105:1464–70. 29863776 10.1002/bjs.10882

[10] Araki K. Pathologic features of superficial esophageal squamous cell carcinoma with lymph node and distal metastasis. Cancer 2002;94:570–5. 11900242 10.1002/cncr.10190

[11] Flejou JF. WHO Classification of digestive tumors: the fourth edition. Ann Pathol 2011;31: (Suppl 5): S27–31. 22054452 10.1016/j.annpat.2011.08.001

[12] Katada C. Esophageal stenosis after endoscopic mucosal resection of superficial esophageal lesions. Gastrointest Endosc 2003;57:165–9. 12556777 10.1067/mge.2003.73

[13] Li Z. Effect of submucosal tunneling endoscopic resection for submucosal tumors at esophagogastric junction and risk factors for failure of en bloc resection. Surg Endosc 2018;32:1326–35. 28812158 10.1007/s00464-017-5810-8

[14] The Paris endoscopic classification of superficial neoplastic lesions: esophagus, stomach, and colon: November 30 to December 1, 2002. Gastrointest Endosc 2003;58: (Suppl 6): S3–43. 14652541 10.1016/s0016-5107(03)02159-x

[15] Shi Q. Tu1325 risk factors for postoperative stricture after endoscopic submucosal dissection for superficial esophageal carcinoma. Gastrointest Endosc 2014;79: ( Suppl 5): AB498. 10.1055/s-0034-1365648 24830402

[16] Broughton GN Janis JE Attinger CE. The basic science of wound healing. Plast Reconstr Surg 2006;117: (Suppl 7): 12S–34S. 16799372 10.1097/01.prs.0000225430.42531.c2

[17] Gotoda T Yamamoto H Soetikno RM. Endoscopic submucosal dissection of early gastric cancer. J Gastroenterol 2006;41:929–42. 17096062 10.1007/s00535-006-1954-3

[18] Chennat J. Complete Barrett's eradication endoscopic mucosal resection: an effective treatment modality for high-grade dysplasia and intramucosal carcinoma – an American single-center experience. Am J Gastroenterol 2009;104:2684–92. 19690526 10.1038/ajg.2009.465

[19] Honda M. Process of healing of mucosal defects in the esophagus after endoscopic mucosal resection: histological evaluation in a dog model. Endoscopy 2010;42:1092–5. 21038294 10.1055/s-0030-1255741

[20] Liu BR. Mucosal loss as a critical factor in esophageal stricture formation after mucosal resection: a pilot experiment in a porcine model. Surg Endosc 2020;34:551–6. 30980136 10.1007/s00464-019-06793-z

[21] Mizuta H. Predictive factors for esophageal stenosis after endoscopic submucosal dissection for superficial esophageal cancer. Dis Esophagus 2009;22:626–31. 19302207 10.1111/j.1442-2050.2009.00954.x

[22] Qumseya B. Predictors of esophageal stricture formation post endoscopic mucosal resection. Clin Endosc 2014;47:155. 24765598 10.5946/ce.2014.47.2.155 PMC3994258

[23] Ono S. Long-term outcomes of endoscopic submucosal dissection for superficial esophageal squamous cell neoplasms. Gastrointest Endosc 2009;70:860–6. 19577748 10.1016/j.gie.2009.04.044

[24] Wang Y Che G. Prognostic value of biomarkers in primary small cell carcinoma of the esophagus. Thorac Cancer 2020;11:1119–20. 32133785 10.1111/1759-7714.13369 PMC7180586

[25] Ceresa IF. Thrombopoietin is not uniquely responsible for thrombocytosis in inflammatory disorders. Platelets 2007;18:579–82. 18041648 10.1080/09537100701593601

[26] Acute cardiovascular care 2014. Eur Heart J Acute Cardiovasc Care 2014;3: (Suppl 2): 01–236. 10.1177/2048872614549721 25342813

[27] Nakamura K Smyth MJ. Targeting cancer-related inflammation in the era of immunotherapy. Immunol Cell Biol 2017;95:325–32. 27999432 10.1038/icb.2016.126

[28] Laplagne C. Latest advances in targeting the tumor microenvironment for tumor suppression. Int J Mol Sci 2019;20:4719. 10.3390/ijms20194719 PMC6801830 31547627

[29] Ishihara R. Endoscopic submucosal dissection for superficial Barrett's esophageal cancer in the Japanese state and perspective. Ann Transl Med 2014;2:24. 25333000 10.3978/j.issn.2305-5839.2014.02.03 PMC4200620

[30] Miwata T. Risk factors for esophageal stenosis after entire circumferential endoscopic submucosal dissection for superficial esophageal squamous cell carcinoma. Surg Endosc 2016;30:4049–56. 26703127 10.1007/s00464-015-4719-3

